# Prevalence and predictors of developing vision-threatening diabetic retinopathy within the first three years of type 2 diabetes

**DOI:** 10.3389/fendo.2023.1305378

**Published:** 2023-12-20

**Authors:** Jia Yan, Bo Li, Ye Chen, Chufeng Gu, Guosheng Dai, Qin Zhang, Zhi Zheng, Dawei Luo, Shuzhi Zhao, Chuandi Zhou

**Affiliations:** ^1^ Department of Ophthalmology, Taizhou Zhangqin Eye Hospital, Taizhou, China; ^2^ Department of Ophthalmology, Shanghai General Hospital, Shanghai Jiao Tong University School of Medicine, National Clinical Research Center for Eye Diseases, Shanghai Key Laboratory of Ocular Fundus Diseases, Shanghai Engineering Center for Visual Science and Photomedicine, Shanghai Engineering Center for Precise Diagnosis and Treatment of Eye Diseases, Shanghai, China; ^3^ Surgical Department, Shanghai General Hospital, Shanghai Jiao Tong University School of Medicine, Shanghai, China

**Keywords:** type 2 diabetes, diabetic retinopathy, vision-threatening diabetic retinopathy, risk factor, early-onset retinopathy

## Abstract

**Purpose:**

To investigate the prevalence of diabetic retinopathy (DR) and vision-threatening DR (VTDR) in patients with type 2 diabetes mellitus (T2DM) stratified by the duration of diabetes and to identify the clinical variations and risk factors for VTDR occurring at different stages of T2DM.

**Methods:**

This was a retrospective comparative study. Patients were divided into short- (≤3 years), intermediate- (3–7 years), and long-duration (>7 years) groups. All patients were followed-up for DR and VTDR development. Risk factors were explored using logistic regression analysis.

**Results:**

A total of,2961 patients were included; among them, 1,036 (35.0%) patients developed DR, and 293 (9.9%) had VTDR. The frequency of VTDR in patients who developed DR in the short-duration group was significantly higher than that in the intermediate-duration group (25.7% vs. 15.0%; p = 0.019), but comparable with that of the long-duration group (25.7% vs. 31.8%; p = 0.138). Patients who developed VTDR within the first 3 years of T2DM were more likely to have a family history of diabetes (p = 0.024), had higher glycated hemoglobin (p = 0.025), were males (p = 0.042), and were notably older at the onset of diabetes (p <0.001) but younger when diagnosed with DR (p <0.001). Moreover, higher glycated hemoglobin (OR = 1.14; 95% CI: 1.00–1.29; p = 0.043) and diabetic nephropathy (DN) (OR = 2.31; 95% CI: 1.08–4.91; p = 0.030) were independent risk factors for developing VTDR during the first 3 years of T2DM.

**Conclusion:**

The risk of DR is not high in persons with ≤3 years’ duration of T2DM, however, if afflicted, the risk of VTDR should never be neglected. More frequent retinal screening is warranted in patients with newly diagnosed T2DM.

## Introduction

Diabetes mellitus is a global epidemic with remarkable morbidity and mortality rates. Globally, the number of people with diabetes is predicted to reach 629 million by 2045, accounting for 9.9% of the global population (1). Diabetic retinopathy (DR), a major microvascular complication of diabetes, is the leading cause of blindness in working-age adults (2). A pooled analysis of 35 population-based studies worldwide (22,896 participants) reported an overall prevalence of 34.6% for any DR and 10.2% for vision-threatening DR (VTDR) (3), including severe non-proliferative DR (NPDR), proliferative DR (PDR), and clinically significant diabetic macular edema (DME) (2). Early detection and intervention of VTDR can prevent up to 98% of visual loss caused by diabetic complications (4). Therefore, the identification of the risk factors for VTDR may assist in the early detection of individuals with the greatest risk.

The risk of DR increases with diabetes duration (5–7). Up to 77.8% of individuals with diabetes for 15 years or more were afflicted with DR (5). The 2000 American Diabetes Association statement suggested that VTDR generally does not affect individuals with type 1 diabetes during the first 3–5 years after diagnosis (8); therefore, an initial retinal examination should be performed within 3–5 years of the initial diagnosis. However, a subsequent study indicated that individuals in whom retinopathy developed during the first 5 years of diabetes had a more rapid progression of retinal pathology, suggesting that dilated eye examinations and retinal photography should be performed from the onset of type 1 diabetes to identify individuals with a high risk of vision-threatening problems (9).

In type 2 diabetes, the thresholds for the prevalence of DR with regard to diabetes duration indicate that the risk of having DR is not linearly associated with exposure to various influencing factors, but the risk relationship may be cumulative, such that the chances of having DR may increase after certain periods of exposure (10). Notably, 20.8% of individuals with type 2 diabetes have DR at the initial diagnosis of diabetes for approximately 4–7 years of undiagnosed diabetes (6). In type 2 diabetes, the first peak for vision-threatening problems caused by retinopathy may occur within the first three years after the diagnosis of type 2 diabetes.

In this study, we analyzed a hospital-based cohort of 2,961 Chinese individuals with type 2 diabetes. Participants were divided into short- (≤3 years), intermediate- (4–7 years), and long-duration (>7 years) groups and were followed up for the development of DR and VTDR. We then focused on the VTDR occurring at each stage of type 2 diabetes regarding its clinical variations and distinct risk factors.

## Materials and methods

### Data collection

The medical charts of patients diagnosed with type 2 diabetes at Shanghai General Hospital from January 2007 to December 2012 were reviewed, and 7,034 patients were identified. Among them, 6,768 underwent standardized eye examinations at baseline. In addition, 311 patients were excluded for severe visual impairments other than DR, such as neovascular age-related macular degeneration, ischemic retinal vein occlusion, uveitis, primary glaucoma, and no light perception in one or both eyes. DR was established according to an outpatient diagnosis based on two or more follow-up visits or a one-time inpatient diagnosis during the exposure period. A total of 3,267 patients were matched in our database, and they had type 2 diabetes for 1.0–46.0 years. However, 306 individuals were excluded because of incomplete data collection, leaving 2,961 individuals eligible for this study.

We explained the purpose of this study to patients with type 2 diabetes and suggested follow-up time points. All patients voluntarily participated in this study without any additional compensation. Oral informed consent was obtained from all patients. The Declaration of Helsinki was followed in this study. Our study was approved by the institutional review board of the Shanghai General Hospital, Shanghai Jiao Tong University School of Medicine.

Screening tests for DR included slit-lamp examinations and color fundus photography through a dilated pupil (Carl Zeiss Meditec AG, Jena, Germany) and optical coherence tomography (OCT) (Heidelberg Engineering, Heidelberg, Germany), if necessary. DR was considered as the presence of any lesion defined by the Early Treatment Diabetic Retinopathy Study (ETDRS). (11) :retinal microaneurysms, blot hemorrhages, hard or soft exudates, venous beading, intraretinal microvascular abnormalities, retinal neovascularization, laser scatter photocoagulation scars, preretinal or vitreous hemorrhage, proliferative membrane and tractional retinal elevation. The primary outcome was the initial occurrence of DR, and the severity of DR was scaled according to the ETDRS grading standards: mild-moderate NPDR (ETDRS level 20–47), severe NPDR (ETDRS level 53), and PDR (ETDRS level ≥60) (11). DME was graded according to the International Clinical Diabetic Retinopathy/Macular Edema Severity Scale (12). Clinically significant DME was defined as retinal edema or hard exudates approaching or involving the fovea. The patients were grouped based on their worse eye into three classes: no DR, mild DR, and VTDR. Mild DR was considered as mild-moderate NPDR, whereas VTDR was defined as the presence of severe NPDR, PDR and/or clinically significant DME. At least two vitreoretinal specialists assessed the retinal lesions per patient. The consistency between the first-round graders was 94.29%. A third-grader, who was not involved in the initial assessment, was asked to deliberate on discrepancies.

The baseline was set as the date of the first type 2 diabetes registration. Clinical details before referral were retrieved for review if the patient had established this diagnosis elsewhere. The date of the initial DR diagnosis was documented. The period between the diagnosis of diabetes and DR was calculated. Individuals with DR were stratified into three groups according to the period clinically free of DR after the diagnosis of type 2 diabetes: early- (≤3 years), intermediate- (4–7 years), and late-onset (>7 years). Individuals who did not have DR during the exposure time were considered censored, and the date of the last visit was recorded.

The data collected included patient demographics, clinical characteristics, and final outcomes at follow-up. The demographics included sex, age at onset of type 2 diabetes and DR, and a family history of type 2 diabetes. The clinical characteristics obtained from the electronic chart records included systolic and diastolic blood pressure, body mass index (BMI), insulin use, and biochemical laboratory information on glycated hemoglobin (HbA1c), fasting glycemia, high-density lipoprotein (HDL) and low-density lipoprotein (LDL) cholesterol, total cholesterol, triglycerides, serum creatinine, and uric acid. The included parameters were assessed every 6–12 months. For individuals with DR, we used the last information before the diagnosis of DR. The last information was carried forward for those who were free of retinopathy.

Apart from DR, all individuals underwent a valid assessment of two other microvascular complications: diabetic nephropathy (DN) and diabetic peripheral neuropathy (DPN). The diagnosis of DN requires at least two albumin excretions of >30 µg/mg creatinine or an excretion rate of >30 mg/24 h on different occasions within one year. DPN was ascertained using biothesiometry measurements with bilateral testing of the big toes. Vibration perception testing (0–50 V) was performed and abnormal readings (on both sides) above age-specific thresholds were recorded (13). After excluding patients with other conditions explaining this neural or renal deficiency, a diagnosis of DN or DPN was established. Only concurrent DN or DPN status may have an impact on the development of DR. Therefore, DN or DPN occurring after the debut of DR is not considered a potential correlate of DR.

### Statistical analysis

Categorical and continuous variables are presented as frequencies (percentages) and medians (interquartile ranges [IQRs]), respectively. Univariate analyses, either ANOVA (continuous factors) or a chi-square test (categorical factors), were used to identify the possible correlates. The significant parameters were then entered into a multivariate logistic regression model as independent variables to explore the independent predictors. Odds ratios (ORs) with 95% confidence intervals (CIs) were also calculated. All analyses were conducted using SPSS (version 21.0; IBM Corp., New York, NY, USA) and two-sided tests with a significance threshold of 0.05.

## Results

In total, 2,961 individuals were included, and the median duration of diabetes was 10.0 years (IQR, 4.0–15.0 years). Among these participants, 660 (22.3%) had diabetes for ≤3 years (short duration group), 529 (17.9%) had diabetes for 4–7 years (intermediate duration group), and 1,772 (59.8%) had diabetes for >7 years (long duration group). There were 1,661 (56.1%) men and 1,300 (43.9%) women, with a median age of 50.0 (IQR, 43.0–57.0) years at diabetes diagnosis. Of 1,036 (35.0%) individuals with DR, 743 (25.1%) had mild DR and 293 (9.9%) had VTDR. The median time interval to DR was 11.0 years (IQR, 6.0–16.0 years), and this period for mild DR and VTDR was 10.0 years (IQR, 6.0–15.0 years) and 13.0 years (IQR, 8.0–17.0 years), respectively. The numbers of individuals with DR were 152 (23.0%), 160 (30.2%), and 724 (40.9%) in the short-, intermediate-, and long-duration groups, respectively. The demographics and concurrent clinical characteristics are presented in **Table 1**.

**Table 1:** Clinical characteristics for 2,961 Chinses patients with type 2 diabetes.

Variables	Total	No DR	Any DR	DR developed in ≤3 years of T2DM	DR developed in 4–7 years of T2DM	DR developed in >7 years of T2DM
	(n = 2,961)	(n = 1,925)	(n = 1,036)	(n = 152)	(n = 160)	(n = 724)
Age at diabetes (y)	50.0 (43.0–57.0)	51.0 (44.0–58.8)	47.0 (41.0–54.0)	51.0 (43.3–57.8)	49.0 (40.3–58.0)	46.0 (40.0–52.0)
Age at DR (y)	/	/	59.0 (52.0–66.0)	52.0 (45.3–59.0)	54.0 (45.2–63.0)	61.0 (55.0–68.0)
Gender						
Male	1,661 (56.1)	1,090 (56.6)	571 (55.1)	98 (64.5)	107 (66.9)	366 (50.6)
Female	1,300 (43.9)	835 (43.4)	465 (44.9)	54 (35.5)	53 (33.1)	358 (49.4)
Family history of diabetes	1,623 (54.8)	1,020 (53.3)	603 (58.2)	92 (60.5)	77 (48.1)	434 (59.9)
Body mass index (kg/m^2^)	24.7 (22.6–27.3)	24.7 (22.6–27.2)	24.7 (22.7–27.3)	24.3 (22.2–27.0)	25.5 (23.5–27.7)	24.6 (22.6–27.2)
SBP (mmHg)	130 (120–140)	130 (120–140)	130 (120–150)	130 (120–140)	130 (120–142)	135 (120–150)
DBP (mmHg)	80 (72–86)	80 (70–85)	80 (75–88)	80 (70–90)	80 (78–90)	80 (75–86)
Glycated hemoglobin (%)	8.4 (7.1–10.1)	8.2 (7.0–9.9)	8.7 (7.4–10.3)	8.6 (7.0–10.5)	8.4 (7.1–10.3)	8.8 (7.6–10.3)
Insulin use	1,648 (55.7)	1,000 (51.9)	648 (62.5)	82 (53.9)	87 (54.4)	479 (66.2)
Total cholesterol (mg/dL)	4.7 (4.0–5.4)	4.7 (4.0–5.3)	4.8 (4.0–5.5)	4.6 (4.0–5.3)	4.7 (4.1–5.5)	4.8 (4.0–5.6)
HDL cholesterol (mg/dL)	1.0 (0.9–1.3)	1.0 (0.9–1.3)	1.1 (0.9–1.3)	1.0 (0.9–1.2)	0.8 (1.0–1.2)	1.1 (0.9–1.3)
LDL cholesterol (mg/dL)	2.8 (2.2–3.4)	2.8 (2.2–3.4)	2.8 (2.3–3.5)	2.7 (2.2–3.3)	2.8 (2.4–3.4)	2.9 (2.3–3.5)
Triglycerides (mg/dL)	1.4 (1.0–2.0)	1.4 (1.0–2.0)	1.4 (0.9–2.0)	1.4 (0.9–2.2)	1.4 (1.0–2.1)	1.3 (0.9–1.9)
Serum creatinine	67.0 (56.0–79.0)	67.0 (56.0–79.0)	66.0 (55.0–79.0)	66.0 (52.5–75.5)	65.0 (58.0–73.0)	66.0 (55.0–81.0)
Uric acid	314 (260–378)	316 (262–380)	312 (258–375)	307 (241–382)	314 (262–383)	313 (259–368)
Urine microalbumin	16.4 (8.4–56.6)	13.9 (7.8–37.9)	23.6 (10.3–131.3)	18.0 (8.8–77.4)	21.9 (10.3–90.5)	25.9 (10.6–151.0)
Diabetic nephropathy	664 (22.4)	371 (19.3)	293 (28.3)	27 (17.8)	45 (28.1)	221 (30.5)
Diabetic peripheral neuropathy	603 (20.4)	318 (16.5)	285 (27.5)	24 (15.8)	33 (20.6)	228 (31.5)

Data are presented as median (interquartile range)/n (%). Symbol "/" means "not applicable".

DR, diabetic retinopathy; T2DM, type 2 diabetes; SBP, systolic blood pressure; DBP, diastolic blood pressure; HDL, high density lipoprotein; LDL, low density lipoprotein.

**Figure 1:**
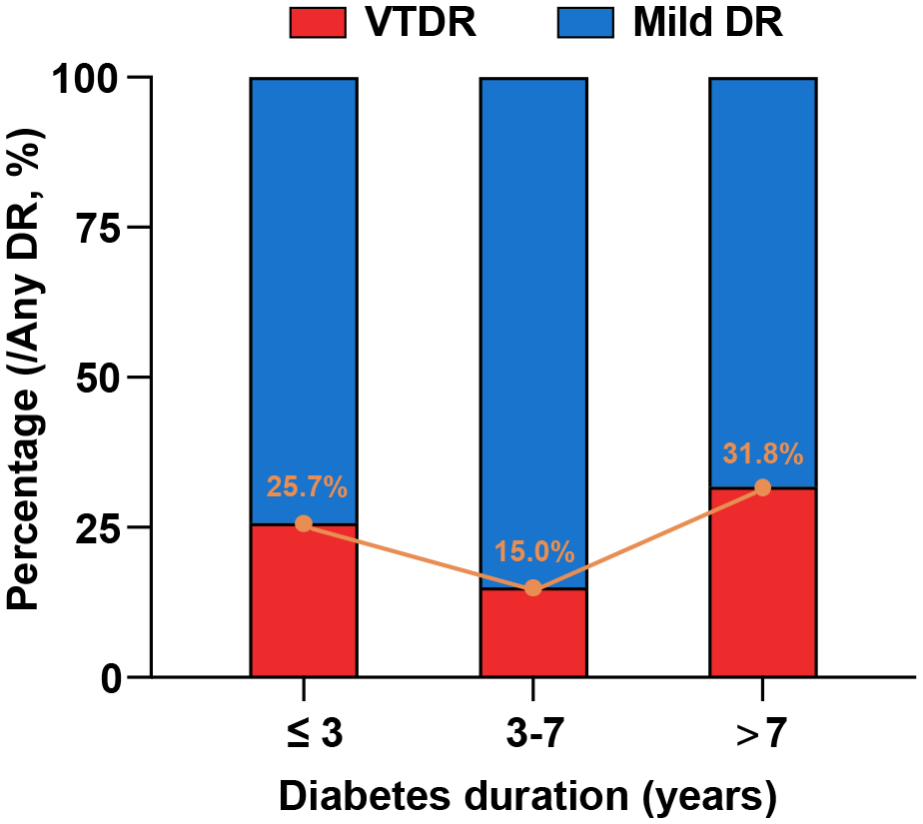
Distribution of different severities of DR stratified by diabetes duration. The proportions of the individuals who developed DR in the short-, intermediate, and long-duration groups were 25.7%, 15.0%, and 31.8%, respectively.

The distribution of the different severities of DR stratified by diabetes duration is shown in **Table 2**; **Figure 1**. Notably, a significantly larger proportion of individuals who developed DR in the short duration group had VTDR (25.7%) than those in the intermediate duration group (15.0%) (p = 0.019). Among those who were diagnosed with DR after 7 years of type 2 diabetes, up to 31.8% of individuals were afflicted with VTDR; however, no significant difference was observed when compared with in the short duration group (p = 0.138).

**Table 2:** The distribution of different severities of diabetic retinopathy stratified by the diabetes duration.

Any DR (n = 1,036)	DR developed in ≤3 years of T2DM	DR developed in 4–7 years of T2DM	DR developed in >7 years of T2DM	p^†^	p^‡^
	(n = 152)	(n = 160)	(n = 724)		
Mild DR (n = 743)	113 (74.3)	136 (85.0)	494 (68.2)	0.019*	0.138
VTDR (n = 293)	39 (25.7)	24 (15.0)	230 (31.8)		

Data are presented as n (%).

DR, diabetic retinopathy; VTDR, vision-threatening diabetic retinopathy; T2DM, type 2 diabetes.

*Statistically significant; ^†^Early vs intermediate; ^‡^Early vs late.

In subsequent analyses, we investigated the clinical variations among the VTDR subgroups (**Table 3**). Those persons who developed VTDR within the first three years were more likely to have a family history of diabetes (66.7% vs. 37.5%, p = 0.024) and higher glycated hemoglobin levels (9.7% vs. 8.3%, p = 0.025) than those who developed VTDR 4–7 years after type 2 diabetes. Furthermore, the persons who developed VTDR within the first three years were notably older at the onset of diabetes (51.8 years vs. 44.0 years, p <0.001) but younger when diagnosed with DR (52.0 years vs. 60.0 years, p <0.001), and were more likely to be males (64.1% vs. 46.5%, p = 0.042) than those who developed VTDR after 7 years of type 2 diabetes.

**Table 3:** Clinical variations of VTDR developed in different stages of T2DM.

Variables	VTDR developed in ≤3 years of T2DM	VTDR developed in 3–7 years of T2DM	VTDR developed in >7 years of T2DM	p^†^	p^‡^
	(n = 39)	(n = 24)	(n = 230)		
Age at diabetes (y)	51.8 (48.0–55.0)	49.0 (44.3–58.3)	44.0 (38.0–50.3)	0.723	<0.001*
Age at DR (y)	52.0 (49.8–57.0)	55.0 (50.0–63.3)	60.0 (53.0–66.6)	0.173	<0.001*
Gender				0.836	0.042*
Male	25 (64.1)	16 (66.7)	107 (46.5)		
Female	14 (35.9)	8 (33.3)	123 (53.5)		
Family history of diabetes	26 (66.7)	9 (37.5)	144 (62.6)	0.024*	0.627
Body mass index (kg/m^2^)	23.6 (21.9–26.8)	26.7 (23.5–28.0)	24.6 (22.5–27.0)	0.067	0.413
SBP (mmHg)	130 (120–140)	130 (125–149)	137 (122–150)	0.810	0.379
DBP (mmHg)	80 (70–90)	80.0 (78–80)	80 (75–90)	0.842	0.930
Glycated hemoglobin (%)	9.7 (8.0–11.7)	8.3 (7.1–10.2)	9.1 (7.8–10.4)	0.025*	0.164
Insulin use	22 (56.4)	14 (58.3)	165 (71.7)	0.911	0.054
Total cholesterol (mg/dL)	4.8 (4.4–5.5)	4.3 (3.9–5.3)	4.9 (4.2–5.8)	0.754	0.533
HDL cholesterol (mg/dL)	1.0 (0.9–1.3)	1.0 (0.9–1.1)	1.1 (0.9–1.3)	0.412	0.232
LDL cholesterol (mg/dL)	2.7 (2.3–3.6)	2.6 (2.3–3.4)	3.0 (2.4–3.6)	0.554	0.434
Triglycerides (mg/dL)	1.2 (0.8–2.9)	1.3 (1.1–1.8)	1.3 (0.9–2.0)	0.972	0.087
Serum creatinine	69.0 (57.0–78.3)	70.0 (58.5–84.0)	68.0 (53.5–84.0)	0.532	0.950
Uric acid	329 (246–397)	322 (275–410)	320 (260–364)	0.362	0.795
Urine microalbumin	105.8 (21.2–381.0)	44.3 (9.0–593.6)	54.3 (14.9–392.6)	0.721	0.866
Diabetic nephropathy	11 (28.2)	7 (29.2)	83 (36.1)	0.935	0.340
Diabetic peripheral neuropathy	7 (17.9)	7 (29.2)	67 (29.1)	0.441	0.148

Data are presented as median (interquartile range)/n(%).

DR, diabetic retinopathy; VTDR, vision-threatening diabetic retinopathy; T2DM, type 2 diabetes; SBP, systolic blood pressure; DBP, diastolic blood pressure; HDL, high density lipoprotein; LDL, low density lipoprotein.

*Statistically significant; ^†^Early vs intermediate; ^‡^Early vs late.

To explore the risk factors for developing VTDR in people with ≤3 years’ type 2 diabetes, univariate and multivariate logistic regressions were performed to determine their independent correlates (**Table 4**). The univariate analysis indicated that higher levels of systolic blood pressure (OR = 1.02; 95% CI: 1.00–1.04; p = 0.029), higher glycated hemoglobin (OR = 1.21; 95% CI: 1.07–1.36; p = 0.002), and the presence of DN (OR = 2.74; 95% CI: 1.31–5.71; p = 0.007) were potential risk factors. Subsequent multivariate analysis demonstrated that higher glycated hemoglobin (OR = 1.14; 95% CI: 1.00–1.29; p = 0.043) and DN (OR = 2.31; 95% CI: 1.08–4.91; p = 0.030) were independent risk factors for the development of VTDR within the first three years of type 2 diabetes.

**Table 4:** Logistic regression for the predictors of developing VTDR within the first 3 years of T2DM.

Variables	Univariate		Multivariate	
	OR (95%CI)	p	OR (95%CI)	p
Age at diabetes (y)	1.00 (0.98–1.03)	0.810		
Age at diabetic retinopathy (y)	1.00 (0.98–1.02)	0.948		
Gender	0.97 (0.49–1.89)	0.918		
Male				
Female				
Family history of diabetes	1.81 (0.91–3.59)	0.089		
Body mass index (kg/m^2^)	0.93 (0.85–1.02)	0.114		
SBP (mmHg)	1.02 (1.00–1.04)	0.029*	1.02 (0.99–1.04)	0.064
DBP (mmHg)	1.00 (0.97–1.03)	0.991		
Glycated hemoglobin (%)	1.21 (1.07–1.36)	0.002	1.14 (1.00–1.29)	0.043
Insulin use	1.32 (0.69–2.53)	0.410		
Total cholesterol (mg/dL)	1.04 (0.82–1.32)	0.720		
HDL cholesterol (mg/dL)	1.84 (0.57–5.94)	0.305		
LDL cholesterol (mg/dL)	1.00 (0.68–1.49)	0.991		
Triglycerides (mg/dL)	1.01 (0.88–1.15)	0.947		
Serum creatinine	1.01 (1.00–1.03)	0.089		
Uric acid	1.00 (0.99–1.00)	0.713		
Diabetic nephropathy	2.74 (1.31–5.71)	0.007*	2.31 (1.08–4.91)	0.030*
Diabetic peripheral neuropathy	1.97 (0.84–4.66)	0.121		

VTDR, vision-threatening diabetic retinopathy; T2DM, type 2 diabetes; OR, odds ratio; CI, confidence interval; SBP, systolic blood pressure; DBP, diastolic blood pressure; HDL, high density lipoprotein; LDL, low density lipoprotein.

*Statistically significant.

We also investigated the factors influencing the development of VTDR in the intermediate- and long-duration groups, the details of which are summarized in **Tables S1**, **S2**. Concurrent DPN was associated with developing VTDR in persons with 4–7 years of type 2 diabetes (OR = 2.52; 95% CI:1.01–6.29; p = 0.048) (**Table S1**). For the VTDR that occurred in the long-duration group, the independent risk factors included younger age at diabetes onset (OR = 0.95; 95% CI:0.92–0.98; p <0.001), female gender (OR = 1.42; 95% CI:1.02–1.96; p = 0.037), higher systolic blood pressure (OR = 1.01; 95% CI: 1.00–1.03; p = 0.014), and higher glycated hemoglobin (OR = 1.12; 95% CI: 1.03–1.22; p = 0.007), insulin use (OR = 1.53; 95% CI:1.09–2.16; p = 0.015), and the presence of DN (OR = 1.46; 95% CI:1.03–2.06; p = 0.034) (**Table S2**).

## Discussion

In this study, we analyzed 2,961 individuals with type 2 diabetes to assess the incidence of DR and VTDR according to diabetes duration. Patients who developed retinopathy within the first three years of diabetes also had a relatively high risk of VTDR. Individuals who developed VTDR within the first three years of type 2 diabetes were more likely to have a family history of diabetes, higher glycated hemoglobin, male sex and were notably older at the onset of diabetes but younger when diagnosed with DR. Concurrent DN and higher glycated hemoglobin levels were predictors of VTDR development during the first three years after type 2 diabetes.

The risk of DR increases with diabetes duration. However, approximately one-fifth of the individuals have DR at the initial diagnosis of type 2 diabetes. Similarly, the incidence of VTDR is not directly proportional to diabetes duration, and varies depending on the severity of retinopathy. The findings of our study suggest that the first three years after diabetes diagnosis is the risk period for developing VTDR for persons with retinal changes. This is consistent with our clinical notion that ocular presentations vary greatly among individuals with newly diagnosed diabetes, most of whom do not have retinopathy; however, if people have diabetic retinal disorders, the initial conditions might have been severe.

DN, an established risk factor for DR development and progression, has been intensively investigated over the past few decades. An 8-year prospective cohort study conducted in 2,135 Chinese people with type 2 diabetes revealed that abnormal renal parameters both at baseline and during the follow-up period, including a high serum creatinine level, low estimated glomerular filtration rate, and high urinary albumin/creatinine ratio, were associated with the development of PDR (14). Elevated expression of vascular endothelial growth factor (VEGF) due to compromised glomerular filtration has been observed in both serum and renal tissues, which might contribute to the development of PDR (15, 16). The same applies to the association among DR, DN, and DPN. Bjerg et al. pointed out that patients with diabetes with any previous microvascular complications had a higher risk of developing further microvascular complications than individuals without any complications (17).

Based on our observations, individuals who developed VTDR within the first three years of type 2 diabetes tended to be older at the onset of diabetes but younger at DR. The ages at diabetes diagnosis were 51.8 years, 49.0 years, and 44.0 years for individuals with VTDR in the short-, intermediate-, and long-duration groups, respectively. Type 2 diabetes is usually insidious at death, and its onset may occur 4–7 years before clinical diagnosis (6, 18). When diabetes is undiagnosed, risk factors for the microvascular disease may be neglected, such as hyperglycemia (19), dyslipidemia (19, 20), microalbuminuria (14), as well as metabolic memory (21), and all of these factors accelerate the development of DR. Consequently, it was not surprising to observe a reversed trend of the age at the diagnosis of DR, in which, the individuals with VTDR were youngest in the short-duration group (52.0 years), followed by 55.0 years in the intermediate-duration group and 60.0 years in the late-duration group. Clearly, the time interval between the diagnosis of type 2 diabetes and DR was shortest for individuals with VTDR in the short duration group. Prior data indicated that it took approximately 5 years from frank diabetes to detectable retinopathy (18). Therefore, the older age at diabetes diagnosis and younger age at DR diagnosis among individuals with VTDR in newly diagnosed type 2 diabetes can be partially explained by delays in seeking care for diabetes.

In this report, individuals with VTDR within the first three years of type 2 diabetes were more likely to be males. This finding is consistent with reports from India (22), England (10, 23, 24), and America (25, 26), which indicated that male sex is an independent risk factor for severe DR in type 2 diabetes, especially near the time of DM diagnosis. However, this relationship appears to weaken with increasing diabetes duration. In our study, females tended to have an increased risk of VTDR 7 years after diabetes. However, the reasons for this sex disparity remain unclear.

Patients who developed VTDR within the first three years were more likely to have a family history of diabetes. This finding suggests that heredity or genetic susceptibility might play a role in the etiology of VTDR in early onset retinopathy (27, 28). Familial patients may be more susceptible to hyperglycemia-induced damage.

This study provided an initial report that depicted an extraordinary accumulation of VTDR during the first three years of diabetes and explored its underlying causes. However, caution should be exercised when interpreting the findings owing to the limitations of this study. First, all participants were recruited from a single center, which might have led to selection bias, and our results may not be generalizable to the entire population of patients with type 2 diabetes. In addition, this study revealed associations, but not causations, owing to its observational design. Furthermore, lifestyle factors, including smoking status, alcohol consumption, sleeping status, and physical activity should also be considered when evaluating the development of DR. However, the strength of this study is the inclusion of a relatively large and homogeneous sample of comprehensive clinical data. The long follow-up time made our results more convincing.

In conclusion, our data provide some general guidance to clinicians that although the risk of having DR is not so high in persons with ≤3 years’ duration of type 2 diabetes; however, if afflicted, the risk of VTDR should never be neglected, especially in patients with compromised glycated hemoglobin and/or DN. Consequently, more frequent retinal screening is warranted in individuals with newly diagnosed diabetes. In addition, individuals who are older at the time of diabetes diagnosis, males, those with a family history of diabetes, and those with compromised glycated hemoglobin may require closer monitoring.

## Data availability statement

The original contributions presented in the study are included in the article/**Supplementary Material**. Further inquiries can be directed to the corresponding authors.

## Ethics statement

The studies involving humans were approved by institutional review board of Shanghai General Hospital, Shanghai Jiao Tong University School of Medicine Identifier: 2022KY024. The studies were conducted in accordance with the local legislation and institutional requirements. Written informed consent for participation in this study was provided by the participants’ legal guardians/next of kin.

## Author contributions

JY: Conceptualization, Data curation, Methodology, Writing – original draft. BL: Validation, Formal analysis, Investigation, Writing – review & editing. YC: Conceptualization, Data curation, Investigation, Writing – review & editing. CG: Writing – original draft, Data curation, Methodology, Validation, Visualization. GD: Writing – original draft. QZ: Writing – original draft. DL: Writing – review & editing. SZ: Conceptualization, Formal analysis, Project administration, Supervision, Writing – review & editing. CZ: Conceptualization, Funding acquisition, Supervision, Writing – review & editing. ZZ: Conceptualization, Project administration, Writing – review & editing.
