# Supplementary material for: Prevalence and predictors of developing vision-threatening diabetic retinopathy within the first three years of type 2 diabetes
Source: Front Endocrinol (Lausanne). 2023 Dec 20;14:1305378. doi: 10.3389/fendo.2023.1305378 (PMC10773727; doi:10.3389/fendo.2023.1305378)
Supplement: Supplementary file 1 [file Table_1.pdf]

Supplementary Material

Prevalence and predictors of developing vision-threatening diabetic retinopathy within the first three years of type 2 diabetes

Jia Yan[1†], Bo Li[2†], Ye Chen[3†], Chufeng Gu[2], Guosheng Dai[1], Qin Zhang[1], Zhi Zheng[2], Dawei Luo[2*], Shuzhi Zhao[2*], Chuandi Zhou[2*]

1. Department of Ophthalmology, Taizhou Zhangqin Eye Hospital, Taizhou, Jiangsu Province, China

2. Department of Ophthalmology, Shanghai General Hospital, Shanghai Jiao Tong University School of Medicine; National Clinical Research Center for Eye Diseases; Shanghai Key Laboratory of Ocular Fundus Diseases; Shanghai Engineering Center for Visual Science and Photomedicine; Shanghai engineering center for precise diagnosis and treatment of eye diseases, 100 Haining Road, Hongkou District, Shanghai, China

3. Surgical Department, Shanghai General Hospital, Shanghai Jiao Tong University School of Medicine 100 Haining Road, Hongkou District, Shanghai, China

Table S1. Logistic regression for the predictors of developing VTDR during 3-7 years of T2DM

Variables	Univariate	
	OR (95%CI)	p
Age at diabetes (y)	1.00(0.96-1.03)	0.760
Age at diabetic retinopathy (y)	0.99(0.96-1.03)	0.712
Gender	0.70(0.29-1.66)	0.414
Male		
Female		
Family history of diabetes	0.54(0.23-1.27)	0.157
Body mass index (kg/m2)	1.02(0.91-1.15)	0.691
SBP (mmHg)	1.01(0.99-1.03)	0.469
DBP (mmHg)	1.00(0.96-1.04)	0.963
Glycated hemoglobin (%)	0.98(0.78-1.23)	0.860
Insulin use	1.58(0.69-3.63)	0.278
Total cholesterol (mg/dL)	1.04(0.68-1.59)	0.848
HDL cholesterol (mg/dL)	0.65(0.11-3.72)	0.628
LDL cholesterol (mg/dL)	1.26(0.77-2.08)	0.361
Triglycerides (mg/dL)	1.02(0.81-1.29)	0.857
Serum creatinine	1.01(0.99-1.02)	0.207
Uric acid	1.00(0.99-1.01)	0.413
Diabetic nephropathy	1.61(0.65-3.98)	0.305
Diabetic peripheral neuropathy	2.52(1.01-6.29)	0.048*

VTDR: vision-threatening diabetic retinopathy; T2DM: type 2 diabetes; OR: odds ratio; CI: confidence interval; SBP: systolic blood pressure; DBP: diastolic blood pressure; HDL: high density lipoprotein; LDL: low density lipoprotein.

*Statistically significant.

Table S2. Logistic regression for the predictors of developing VTDR after 7 years of T2DM

Variables	Univariate		Multivariate	
	OR (95%CI)	p	OR (95%CI)	p
Age at diabetes (y)	0.95(0.94-0.97)	<0.001*	0.95(0.92-0.98)	0.001*
Age at diabetic retinopathy (y)	0.96(0.95-0.98)	<0.001*	1.00(0.97-1.03)	0.910
Gender	1.33(1.01-1.75)	0.046*	1.42(1.02-1.96)	0.037*
Male				
Female				
Family history of diabetes	1.35(1.02-1.80)	0.038*	1.20(0.87-1.65)	0.278
Body mass index (kg/m2)	1.01(0.97-1.05)	0.689		
SBP (mmHg)	1.01(1.00-1.02)	0.003*	1.01(1.00-1.03)	0.014*
DBP (mmHg)	1.02(1.01-1.04)	0.005*	1.00(0.98-1.02)	0.806
Glycated hemoglobin (%)	1.16(1.08-1.25)	<0.001*	1.12(1.03-1.22)	0.007*
Insulin use	1.80(1.33-2.44)	<0.001*	1.53(1.09-2.16)	0.015*
Total cholesterol (mg/dL)	1.24(1.11-1.39)	<0.001*	0.90(0.73-1.12)	0.350
HDL cholesterol (mg/dL)	1.53(1.00-2.34)	0.049*	1.56(0.96-2.53)	0.070
LDL cholesterol (mg/dL)	1.32(1.13-1.54)	0.001*	1.24(0.94-1.63)	0.131
Triglycerides (mg/dL)	0.99(0.88-1.11)	0.846		
Serum creatinine	1.00(0.99-1.01)	0.811		
Uric acid	1.00(0.99-1.00)	0.958		
Diabetic nephropathy	1.72(1.28-2.30)	<0.001*	1.46(1.03-2.06)	0.034*
Diabetic peripheral neuropathy	1.22(0.90-1.66)	0.207		

VTDR: vision-threatening diabetic retinopathy; T2DM: type 2 diabetes; OR: odds ratio; CI: confidence. interval; SBP: systolic blood pressure; DBP: diastolic blood pressure; HDL: high density lipoprotein; LDL: low density lipoprotein.

*Statistically significant.
